# Supplementary material for: Regulation of heterosis-associated gene expression complementation in maize hybrids
Source: Genome Biol. 2025 Sep 22;26:291. doi: 10.1186/s13059-025-03768-3 (PMC12455817; doi:10.1186/s13059-025-03768-3)
Supplement: Supplementary file 4 — Additional file 4: Table S4 Proportion of heterotic variance explained by the number of SPE genes on better-parent heterosis for different root phenotypes. Table S6 Synteny of all active genes with eQTL. [file 13059_2025_3768_MOESM4_ESM.docx]

**Table S4**: Proportion of heterotic variance explained by the number of SPE genes on better-parent heterosis for different root phenotypes

| Trait | B73xIBM-RILs | | | Mo17xIBM-RILs | | |
| --- | --- | --- | --- | --- | --- | --- |
|  | **σ^2^_Het_** | **σ^2^_G_** | **p_Het_** | **σ^2^_Het_** | **σ^2^_G_** | **p_Het_** |
| No. Of root tips | 0.144 | 0.204 | 29% (0.291) | 0.317 | 0.312 | -2% (-0.015) |
| Total root volume | 1.834 | 2.277 | 19% (0.194) | 1.159 | 1.362 | 15% (0.149) |
| Total root length | 1.830 | 2.452 | 25% (0.254) | 4.005 | 4.190 | 4% (0.044) |
| Lateral root density | 6.804 | 7.908 | 14% (0.140) | 10.938 | 10.490 | -4% (0.043) |

σ^2^_Het_ = unexplained genetic variance of heterosis effect, not associated with SPE genes; σ^2^_G_ = total genetic variance among the hybrid genotypes; p_Het_ = Coefficient of determination: proportion of the heterotic variance explained by the number of SPE genes.

**Table S6**: Synteny of all active genes with eQTL

| **Regulation** | **Syntenic** | **Non-syntenic** |
| --- | --- | --- |
| **Genes with *cis*-eQTL** | 9292 (78%) | 2694 (22%) |
| **Genes with *trans*-eQTL** | 496 (30%) | 1131 (70%) |
